# Supplementary material for: Synthetic lethality between the cohesin subunits STAG1 and STAG2 in diverse cancer contexts
Source: eLife. 2017 Jul 10;6:e26980. doi: 10.7554/eLife.26980 (PMC5531830; doi:10.7554/eLife.26980)
Supplement: Supplementary file 1. — DOI: http://dx.doi.org/10.7554/eLife.26980.019 [file elife-26980-supp1.docx]

**Supplementary file 1**

**sgRNA sequences used in this study.**

| sgRNA name | Sequence | Use |
| --- | --- | --- |
| sgRNA 505 | TGGAAAACGAGCCAATGAG | *STAG2* inactivation |
| sgRNA 502 | TTTCGACATACAAGCACCC | *STAG2* inactivation |
| sgRNA STAG2_16 | GATTTGAACTTCTTCCACTG | *STAG2* inactivation and competition assay |
| sgRNA STAG2_ 19 | GGAAAACGAGCCAATGAG | *STAG2* inactivation and competition assay (control sgRNA KBM-7) |
| STAG1_3 | GTCTGACAAACCCGTCAAAA | Competition assay |
| STAG1_18 | GAATCATATAAACAAGACA | Competition assay |
| STAG2_5 | GAACTCCTGCTACAAAAG | Competition assay |
| STAG2_16 | GATTTGAACTTCTTCCACTG | Competition assay |
| RAD21_2 | GATCGTGAGATAATGAGAGA | Competition assay |
| SMC3_9 | GGATGCAAGAGATAAAATGG | Competition assay |
| CTRL | GTAGCGAACGTGTCCGGCGT | Competition assay (control sgRNA HCT 116) |
