# Supplementary material for: Synthetic lethality between the cohesin subunits STAG1 and STAG2 in diverse cancer contexts
Source: eLife. 2017 Jul 10;6:e26980. doi: 10.7554/eLife.26980 (PMC5531830; doi:10.7554/eLife.26980)
Supplement: Supplementary file 2. — Sources, STAG2 status and authentication information (STR fingerprinting) of cell lines are listed. DOI: http://dx.doi.org/10.7554/eLife.26980.020 [file elife-26980-supp2.docx]

**Supplementary file 2**

**Cell lines used in this study.**

| Cell line | Cancer | STAG2 status | Reference for STAG2 status | Source | STR confirmed |
| --- | --- | --- | --- | --- | --- |
| HCT 116 parental | Colon | Wild-type | (Solomon et al., 2011), this study | ATCC | Yes |
| HCT 116 STAG2- 502c1 | Colon | M255fs (CRISPR KO) | This study | This study | Yes |
| HCT 116 STAG2- 502c4 | Colon | T220fs (CRISPR KO) | This study | This study | Yes |
| HCT 116 S97X | Colon | S97* (engineered) | (J. S. Kim et al., 2016) | (J. S. Kim et al., 2016) | Yes |
| HCT 116 I885fs | Colon | I885fs (engineered) | (J. S. Kim et al., 2016) | (J. S. Kim et al., 2016) | Yes |
| HCT 116 Y1142fs | Colon | Y1142fs (engineered) | (J. S. Kim et al., 2016) | (J. S. Kim et al., 2016) | Yes |
|  |  |  |  |  |  |
| KBM-7 parental | Leukemia | Wild-type | This study | Horizon | Yes |
| KBM-7 *STAG2*- 16_19c12 | Leukemia | P166fs (CRISPR KO) | This study | This study | Yes |
|  |  |  |  |  |  |
| hTERT RPE-1 | Normal retinal pigment epithelial cells | Wild-type | This study | ATCC | Yes |
|  |  |  |  |  |  |
| 5637 | Bladder | Expressed | (Solomon et al., 2013) | ATCC | Yes |
| 639-V | Bladder | Expressed | (Balbas-Martinez et al., 2013) | DSMZ | Yes |
| 647-V | Bladder | Expressed | (Solomon et al., 2013) | DSMZ | Yes |
| J82 | Bladder | Expressed | (Solomon et al., 2013) | ATCC | Yes |
| JMSU-1 | Bladder | Wild-type | (Solomon et al., 2013) | DSMZ | Yes |
| KU-19-19 | Bladder | Expressed | (Solomon et al., 2013) | DSMZ | Yes |
| RT4 | Bladder | Expressed | (Solomon et al., 2013) | ATCC | Yes |
| T24 | Bladder | Expressed | (Solomon et al., 2013) | ATCC | Yes |
| UM-UC-5 | Bladder | Expressed | (Balbas-Martinez et al., 2013) | H. B. Grossman (MDACC, Houston) | Yes |
| UM-UC-5 wt | Bladder | Expressed | This study | This study | Yes |
| UM-UC-18 | Bladder | Expressed | (Balbas-Martinez et al., 2013) | H. B. Grossman (MDACC, Houston) | Yes |
| LGWO1 | Bladder | Expression silenced | (Balbas-Martinez et al., 2013) | J. Reeder (Rochester, New York) | Yes |
| MGH-U3 | Bladder | Expression silenced | (Balbas-Martinez et al., 2013) | F. Radvanyi (Institut Curie, Paris, France) | Yes |
| UM-UC-5 c6 | Bladder | R261fs and E262* (CRISPR KO) | This study | This study | Yes |
| UM-UC-3 | Bladder | F983fs | (Solomon et al., 2013) | ATCC | Yes |
| UM-UC-6 | Bladder | R305* | (Balbas-Martinez et al., 2013) | H. B. Grossman (MDACC, Houston) | Yes |
| VM-CUB-3 | Bladder | S97fs | (Balbas-Martinez et al., 2013; Solomon et al., 2013) | L. J. Old (Memorial Sloan-Kettering Cancer Center, NY) | Yes |
|  |  |  |  |  |  |
| A-673 | Ewing sarcoma | Wild-type | (Solomon et al., 2011; Tirode et al., 2014) | ATCC | Yes |
| TC-71 | Ewing sarcoma | Wild-type | (Solomon et al., 2011; Tirode et al., 2014) | Timothy Triche (Children’s Hospital, Los Angeles) | Yes |
| SK-N-MC | Ewing sarcoma | M1_R546Del | (Tirode et al., 2014) | June Biedler (Memorial Sloan Kettering Cancer Center, NY) | Yes |
| TC-32 | Ewing sarcoma | Y636fs | (Solomon et al., 2011) | Timothy Triche (Children’s Hospital, Los Angeles) | Yes |
